# Supplementary material for: The bidirectional association between depressive symptoms, assessed by the HADS, and albuminuria–A longitudinal population-based cohort study with repeated measures from the HUNT2 and HUNT3 Study
Source: PLoS One. 2022 Sep 15;17(9):e0274271. doi: 10.1371/journal.pone.0274271 (PMC9477298; doi:10.1371/journal.pone.0274271)
Supplement: S1 File — (DOCX) [file pone.0274271.s001.docx]

| S Fact box 1 Overview of statistical models | |
| --- | --- |
| Hypotheses 1 (H1): Depression symptoms in HUNT2 (exposure) are associated with albuminuria levels in HUNT3 (outcome). | |
| Statistical formula, Equation 1 (Eqn1):  $\gamma_{i}=\beta_{0}+{\beta_{Sex}X}_{i,Sex}+{\beta_{Age}X}_{i,Age}+{{\beta_{k}X}_{i,k}+\varepsilon}_{i}$ | Explanation of the statistical formula  _i_ = individual participant  γ_i_ = HADS score in HUNT3 for individual *i*  β_0_ = intercept with albuminuria levels in HUNT2  *k* = confounder variables (including HADS score in HUNT2)  ε_i_ is the residual effect |
| Hypothesis 2 (H2): Albuminuria levels (exposure) in HUNT2 are associated with depression symptoms in HUNT3 (outcome). | |
| Statistical formula, Equation 2 (Eqn2):  $z_{i}=\beta_{0}+{\beta_{Sex}X}_{iSex}+{\beta_{Age}X}_{iAge}+\ldots+{{\beta_{k}X}_{i,k}+\varepsilon}_{i}$ | Explanation of the statistical formula  _i_ = individual participant  z_i_ = albuminuria level for individual *i* in HUNT3.  β_0_ = intercept with depression symptoms in HUNT2  *k* = confounder variables (including albuminuria levels in HUNT2)  ε_i_ is the residual effect |
