# Supplementary material for: The bidirectional association between depressive symptoms, assessed by the HADS, and albuminuria–A longitudinal population-based cohort study with repeated measures from the HUNT2 and HUNT3 Study
Source: PLoS One. 2022 Sep 15;17(9):e0274271. doi: 10.1371/journal.pone.0274271 (PMC9477298; doi:10.1371/journal.pone.0274271)
Supplement: S1 Table — (DOCX) [file pone.0274271.s002.docx]

| **S Table 1. General linear regression results in complete data set** | | |
| --- | --- | --- |
|  | **Albuminuria HUNT3 (log)^1^**  Model 3^3^ | **HADS-Depression H3 (+1)^2^**  Model 3^3^ |
| Variable | β (95% CI) | β (95% CI) |
| Intercept | -2.26 (-3.12, -1.41)*** | .03 (-1.34,1.95) |
| Albuminuria H2 (mg/mmol) | .036 (.03, .04)*** | -.005(-.02,.02) |
| Age (years) | .03 (.02,.03)*** | .01 (.001,.03)* |
| Male sex | -.04 (-.21,.14) | .07 (-.28,.40) |
| HADS- Depression H2 | -.002 (-.02,.01) | .54 (.50,.59)*** |
| Education (in years) (reference) |  |  |
| 10-12 years | .12 (.01, .23)* | -.23 (-.46,.009) |
| >12 years | .14 (.003, .27)* | -.26 (-.54,.02) |
| Body mass index (kg/m^2^) | .003 (-.01,.01) | .04 (.01,.06)** |
| Smoking status (reference) |  |  |
| Previous | .06 (-.04,.17) | -.02 (-.25,.20) |
| Current | .24 (.11, .38)*** | .41 (.12,.71)** |
| Cholesterol (mg/mmol) | -.01 (-.05,.03) | .04 (-.05,.13) |
| Blood Pressure medication (yes) | .17 (.06, .29)** | .17 (-.07,.41) |
| CVD (yes) | .005 (-.15,.16) | .13 (-.22,.50) |
| eGFR (CKD-epi) | .006 (-.0002,.01). | .008 (-.003.02) |
| Diabetes (yes) | .37 (.21, .52)*** | .30(-.02,.64) |
| Systolic blood pressure (mmHg) | .003 (.0001, .005)* | -.0008(-.006,.005) |
| Alcohol status (units per week) |  |  |
| 1-7 | -.01 (-.12,.09) | -.29 (-.52,-.07)* |
| 8-14 | -.06 (-.23,.11) | -.45 (-.78,-.10)** |
| >15 | -.12 (-.43,.18) | -.5(-1.04,.14) |
| Statistical tests | R^2^ (multiple)= .15  F = 16.42, df (28,1674),  p <0.001  Residual standard error = .96 with df=1674  1257 observations deleted due to missingness | † Pseudo R^2^ = .29, p-value = 0.00  Dispersion parameter for Gamma family = 0.32  832 observations deleted due to missingness |
| ^1^Albuminuria is measured by Albumin Creatinine Ratio (ACR) in urine in mg/mmol. The ACR was treated as a continuous variable and log-transformed and were fitted with a linear model with a Gaussian distribution.  ^2^A value of 1 was added to the score as gamma distribution does not exist for 0, and the depression subscale of the Hospital Anxiety and Depression Scores include the value 0.  ^3^ Model 3 included the confounders age, sex, baseline level of the outcome variable, education, body mass index (BMI), smoking, cholesterol, eGFR, diabetes, systolic blood pressure (SBP), blood pressure medication and alcohol.  All the predictors, k, in the model were measured at HUNT2. The grey area in the table indicates the results for the covariates (k) in the model and is not the focus of this article.  † For the GLM with depression (+1) at HUNT2 as response, a pseudo R^2^ is calculated as 1-(residual deviance/null deviance), here giving a value of 1 -(654.42 /924.73) = .29, with df = 2099 for the residual deviance and df = 2117 for the null deviance. P-value is calculated from a deviance goodness of fit test.  R2 is the coefficient of determination. | | |
