# Supplementary material for: The bidirectional association between depressive symptoms, assessed by the HADS, and albuminuria–A longitudinal population-based cohort study with repeated measures from the HUNT2 and HUNT3 Study
Source: PLoS One. 2022 Sep 15;17(9):e0274271. doi: 10.1371/journal.pone.0274271 (PMC9477298; doi:10.1371/journal.pone.0274271)
Supplement: S2 Table — (DOCX) [file pone.0274271.s003.docx]

| **S Table 2.**  **Analysis of Variance (ANOVA) for the covariates in HUNT2 explaining Albuminuria or depression in HUNT3** | | | | | | |
| --- | --- | --- | --- | --- | --- | --- |
|  | **Albuminuria H3 (log)^1^**  **Model 3^2^** | | | **HADS-Depression H3 (+1)^2^**  **Model 3^2^** | | |
| **Variable** | **SSQ** | **F (df1,df2)** | **Partial eta^2^** | **SSQ** | **F (df1,df2)** | **Partial eta^2^** |
| Age | 190.70 | 114.82 (1, 115.96)*** | .06 | 590.97 | 89.31 (1, 4360.91)*** | .03 |
| Sex | 12.69 | 8.65 (1, 207.33)** | .004 | 82.90 | 11.83 (1, 1570.64) *** | .005 |
| Albuminuria H2 | 111.67 | 80.09 (1, 225.78)*** | .04 | 1.28 | .10( 1, 3092.15) | .00007 |
| Depression H2 | 1.25 | .36 (1, 126.87) | .0004 | 6748.28 | 909.07 (1, 673.11)*** | .27 |
| Education level | 2.74 | .89 (2, 422.66) | .001 | 259.35 | 18.47 (2, 1308.68)*** | .01 |
| Body Mass Idex | 5.70 | 4.20 (1, 355.68)* | .002 | 178.22 | 26.12 (1, 2262.75)*** | .01 |
| Smoking status | 17.95 | 5.82 (2, 166.05)** | .006 | 144.25 | 7.76 (2, 181.84)*** | .008 |
| Cholesterol | 1.32 | .77(1, 354.37) | .0005 | 1.52 | .15 (1, 4059.47) | .00008 |
| Blood Pressure medication | 9.53 | 5.58 (1, 131.28)* | .003 | 51.61 | 7.84 (1, 6371.19)** | .003 |
| Cardiovascular disease | .52 | .24(1, 603.76) | .0002 | 22.08 | 3.40 (1, 13065.78) | .001 |
| Kidney function | 1.72 | .81 (1, 177.38) | .0006 | 12.37 | 1.65 (1, 1486.35) | .0007 |
| Diabetes | 41.62 | 25.43 (1, 100.79)*** | .01 | 9.76 | 1.43 (1, 5818.49) | .0005 |
| Systolic blood pressure | 5.30 | 2.82 (1, 122.99) | .002 | 15.66 | 2.22 (1, 2591.61) | .0009 |
| Alcohol | 2.04 | .21 (3, 222.56) | .0007 | 139.34 | 6.85 (3, 2686.15)*** | .008 |
| Residual | 2889.62 |  |  | 18101.17 |  |  |
| ^1^Albuminuria is measured by Albumin Creatinine Ratio (ACR) in urine in mg/mmol. The ACR was treated as a continuous variable and log-transformed and were fitted with a linear model with a Gaussian distribution.  ^2^Depression symptoms is measured by the Hospital Anxiety and Depression Scale. A value of 1 was added to the score as gamma distribution does not exist for 0, and HADS scores include the value 0.  SSQ is the sums of squares. F is the F statistics with the degrees of freedom (df)  ^2^Model 3 included the confounders age, sex, baseline level of the outcome variable, education, work level, BMI, smoking, cholesterol, eGFR, diabetes, SBP, and alcohol. | | | | | | |
