# Supplementary material for: The bidirectional association between depressive symptoms, assessed by the HADS, and albuminuria–A longitudinal population-based cohort study with repeated measures from the HUNT2 and HUNT3 Study
Source: PLoS One. 2022 Sep 15;17(9):e0274271. doi: 10.1371/journal.pone.0274271 (PMC9477298; doi:10.1371/journal.pone.0274271)
Supplement: S3 Table — (DOCX) [file pone.0274271.s004.docx]

| **S Table 3**  **Sensitivity analysis for the imputed data for model 3 with depression for HUNT 3 as response** | | |
| --- | --- | --- |
|  | **Model 3 for Depression HUNT 3** | |
| **Analysis** | β_A_ (95% CI) | p-value |
| Complete case | -0.005 (-.02,.02) | .59 |
| Sensitivity (MI δ = 0 )/ Primary (MI MAR) | -0.0012 (-.02,.02) | .90 |
| Sensitivity (MI δ = 1 ) | -0.0020 (-.02,.02) | .84 |
| Sensitivity (MI δ = 2 ) | -0.0034 (-.02,.02) | .74 |
| Sensitivity (MI δ = 3 ) | -0.0035 (-.02,.02) | .73 |
| Sensitivity (MI δ = 4 ) | -0.0044 (-.03,.02) | .67 |
| Sensitivity (MI δ = 5 ) | -0.0064 (-.03,.01) | .54 |
| β_A_ is the estimated regression coefficient for albuminuria in HUNT2 when depression symptoms in HUNT3 is the response, SE are the standard error of the estimate, p-value are the calculated p-value for the estimate.  We note that specifying an adjustment in HADS for HUNT 2 and HUNT3 only, and since it is used to impute other incomplete values, they can affect imputations in these (the effect of this depends on the correlation between HADS for HUNT 2 and HUNT3 and the other variables). | | |
