# Supplementary material for: The bidirectional association between depressive symptoms, assessed by the HADS, and albuminuria–A longitudinal population-based cohort study with repeated measures from the HUNT2 and HUNT3 Study
Source: PLoS One. 2022 Sep 15;17(9):e0274271. doi: 10.1371/journal.pone.0274271 (PMC9477298; doi:10.1371/journal.pone.0274271)
Supplement: Attachment — Submitted filename: PONE_D_22_08418_2022.docx [file pone.0274271.s005.docx]

**A BIDIRECTIONAL ASSOCIATION STUDY BETWEEN DEPRESSIVE SYMPTOMS AND ALBUMINURIA – A LONGITUDINAL POPULATION-BASED COHORT WITH REPEATED MEASURES FROM THE HUNT2 AND HUNT3 STUDY**

**General comments**

This paper presents findings on important areas such as depression, albuminuria, and CVD. The use of cohort compared to cross-sectional studies helps improve the scant knowledge available on the hypothesis generated in the study. The use of robust statistical tools is commendable.

The article will benefit from proof reading to correct a few grammar and typographical errors.

**Introduction**

The introductory section will benefit from clearly defining the purpose of the study. Authors begin by linking CVD to depression and albuminuria which is fine. Then this is lost and a focus is now on the bidirectional relationship between depression and albuminuria.

I do understand that the article hinges on the bidirectional relationship between albuminuria and depression and this was emphasized in paragraphs 2 and 3 of the introductory section.

In the second paragraph, authors stressed on the fact that only one study has used a cohort, then they introduced a cross-sectional cohort study in the third paragraph. They also make the point that this is the first cohort study looking at the bidirectional relationship between depression and albuminuria. I suggest, authors clarify this and/or make this important point clear as it provides a good strength for the paper.

**Methods**

I have no comment on this section

**Results**

Authors report no significant association between depression levels and albuminuria and vice versa but report a that some small percentage of the relationship is also attributable to depression and to albuminuria. This sounds confusing, can authors clarify these statements.

Table 2 should be improved by creating a heading to show what r, what is in the bracket and the p value stands for or can be explained at the foot note.

**Discussion**

Again, authors repeat this statement “For albuminuria, only 0.04% is explained by previous depression levels, and for depression symptoms, only 0.007% is explained by previous albuminuria levels”. Ideally, there should first be an association and then we go and look for the “weight” influence of the explanatory variables.

Comparison of studies which used proteinuria to current study which used albuminuria is a little out of place. Especially, when authors have hammered on its limitations in comparison to albuminuria and that is the more reason why they are using albuminuria. Is it possible to find more suitable articles to support their discussions?

The last sentence in paragraph 3 is incomplete. Authors should kindly resolve this since it was addressing an important point

**Conclusion**

In the conclusion, authors report no association between depression levels and albuminuria and vice versa. However, authors failed to provide guidance on the next action or way forward.
