# Supplementary material for: The bidirectional association between depressive symptoms, assessed by the HADS, and albuminuria–A longitudinal population-based cohort study with repeated measures from the HUNT2 and HUNT3 Study
Source: PLoS One. 2022 Sep 15;17(9):e0274271. doi: 10.1371/journal.pone.0274271 (PMC9477298; doi:10.1371/journal.pone.0274271)
Supplement: Attachment — Submitted filename: Rebuttal letter PONE_D_22_08418_2022.docx [file pone.0274271.s006.docx]

To the attention of Plos One	8 Aug 2022

Academic Editor PLOS ONE
Angela Lupattelli, PhD

**Major revision of manuscript PONE-D-22-08418**

“A bidirectional association study between depressive symptoms and albuminuria – A longitudinal population-based cohort with repeated measures from the HUNT2 and HUNT3

We would like to thank the editor and the reviewers for putting in time and effort to elevate the quality of our manuscript. All comments have been addressed and revisions to the manuscripts have been applied accordingly.

Point-by-point responses are provided below with statements to where (section, paragraph and sentence) the amendments are made in the manuscript. The reviewers’ concerns are in black font and our response are in blue font.

The sentences we added to the paper is marked with quote marks and italic font in this revision letter “…”: In the manuscript the changes are marked with track changes in colors in the marked version. None of the co-authors have a conflict regarding funder-policies.

Sincerely,

Lise Tuset Gustad

E-mail: lise.t.gustad@nord.no	Levanger, 12 Aug 2022

Journal Requirements:

Authors reply; All editors concerns and journal requirements are answered in the cover letter

6. Review Comments to the Author

Please use the space provided to explain your answers to the questions above. You may also include additional comments for the author, including concerns about dual publication, research ethics, or publication ethics. (Please upload your review as an attachment if it exceeds 20,000 characters)

Reviewer #1: This is an interesting paper, in which the bidirectional relation between albuminuria and depressive symptoms as measured by HADS was investigated in a longitudinal way. Unlike some cross sectional studies, no clinically relevant association was observed.
The study is well performed and the paper is well written. I have no major remarks, but given the discrepancy between studies that used different ways to assess depressive symptoms (e.g. HADS and mini International Neuropsychiatric Interviews. To which extent do these scales measure the same or different constructs? This would be relevant to know for the reader in order to interprete these discrepancies.

Authors response: In the discussion we now incorporate more detailed discussions about the differences between studies that might explain the conflicting results.

In the title "depressive symptoms by HADS"" might be the most appropriate term

Authors response: We changed the title according to reviewers suggestions and the new title reads*; “*The bidirectional association between depressive symptoms, assessed by the HADS and albuminuria – A longitudinal population-based cohort with repeated measures from the HUNT2 and HUNT3 Study”

Reviewer #2: A BIDIRECTIONAL ASSOCIATION STUDY BETWEEN DEPRESSIVE SYMPTOMS AND ALBUMINURIA – A LONGITUDINAL POPULATION-BASED COHORT WITH REPEATED MEASURES FROM THE HUNT2 AND HUNT3 STUDY
General comments
This paper presents findings on important areas such as depression, albuminuria, and CVD. The use of cohort compared to cross-sectional studies helps improve the scant knowledge available on the hypothesis generated in the study. The use of robust statistical tools is commendable.
The article will benefit from proof reading to correct a few grammar and typographical errors.

Authors response: MB did the final English proofreading This is now stated in our manuscript:

*“MB did the final English proofreading”(authors contributions)*

Introduction
The introductory section will benefit from clearly defining the purpose of the study. Authors begin by linking CVD to depression and albuminuria which is fine. Then this is lost and a focus is now on the bidirectional relationship between depression and albuminuria.
I do understand that the article hinges on the bidirectional relationship between albuminuria and depression and this was emphasized in paragraphs 2 and 3 of the introductory section.

*Authors response:* We have reorganized the introductory section in order to make our aim more clear.

In the second paragraph, authors stressed on the fact that only one study has used a cohort, then they introduced a cross-sectional cohort study in the third paragraph. They also make the point that this is the first cohort study looking at the bidirectional relationship between depression and albuminuria. I suggest, authors clarify this and/or make this important point clear as it provides a good strength for the paper.

*Authors response*: We understand that we have not been clear about cross-sectional and longitudinal studies. We hope that the reorganization of the introduction described above is satisfactory in solving this issue as well. Since we sent in our manuscript one new longitudinal study have been published that has measured albuminuria and depression repeatedly; published in 2022 by Liu et al [1], which we have included in the manuscript. That paper used the Center for Epidemiologic Studies Depression Scale (-CES-D),and did not, like us, find a convincing longitudinal relation between albuminuria and depression. The HR (95% confidence interval) for depression with UACR ≥30 mg/g compared to those with UACR <10 mg/g was 1.25 (95% CI, 0.94 to 1.65). Our study thus confirm the uncertainty related to albuminuria being a useful biomarker to albuminuria and still our study is the first study to assess the usefulness to use albuminuria as a biomarker for depression.

*…*
Methods
I have no comment on this section

Results
Authors report no significant association between depression levels and albuminuria and vice versa but report a that some small percentage of the relationship is also attributable to depression and to albuminuria. This sounds confusing, can authors clarify these statements.

*Authors respons;* We agree that this was confusing and have now altered the statements. We hope the new statement is better understood. The paragraph now reads;

*“Even though albuminuria in HUNT2 was significantly associated with HADS-D symptoms in HUNT3, the correlation coefficient of 0.07 indicates a negligible association between the two. This corresponds to albuminuria explaining only 0.007% of the variance in depression levels in HUNT3. Thus, albuminuria is probably not a useful biomarker for future depression symptoms. HADS-D in HUNT2 had no significant correlation with albuminuria in HUNT3, with a correlation coefficient of 0.04 for HADS-D7 and 0.03 for HADS-D4. With wide confidence intervals including the null, 0.04% of the variance in HUNT 3 albuminuria levels was explained by previous depression levels, making depression symptoms as measured by HADS ineffectual as a biomarker for albuminuria” Discussion page 24.*

*Authors reply;*
Table 2 should be improved by creating a heading to show what r, what is in the bracket and the p value stands for or can be explained at the foot note.

Authors response: We have rearranged our table footnotes in order to put the explanations of our abbreviations more central

“*Abbreviations: r =correlation coefficient, ()=(standard error of pooled estimates), p= p-value.”*

Discussion
Again, authors repeat this statement “For albuminuria, only 0.04% is explained by previous depression levels, and for depression symptoms, only 0.007% is explained by previous albuminuria levels”. Ideally, there should first be an association and then we go and look for the “weight” influence of the explanatory variables.

Authors reply: We have rephrased this part of the discussion. It now reads;

*“Even though albuminuria in HUNT2 was significantly associated with HADS-D symptoms in HUNT3, the correlation coefficient of 0.07 indicates a negligible association between the two. This corresponds to albuminuria explaining only 0.007% of the variance in depression levels in HUNT3. Thus, albuminuria is probably not a useful biomarker for future depression symptoms. HADS-D in HUNT2 had no significant correlation with albuminuria in HUNT3, with a correlation coefficient of 0.04 for HADS-D7 and 0.03 for HADS-D4. With wide confidence intervals including the null, 0.04% of the variance in HUNT 3 albuminuria levels was explained by previous depression levels, making depression symptoms as measured by HADS ineffectual as a biomarker for albuminuria”. (Discussion page 24)*

Comparison of studies which used proteinuria to current study which used albuminuria is a little out of place. Especially, when authors have hammered on its limitations in comparison to albuminuria and that is the more reason why they are using albuminuria. Is it possible to find more suitable articles to support their discussions?

Since we sent in our manuscript one new longitudinal study have been published that has measured albuminuria and depression repeatedly; published in 2022 by Liu et al [1], we have now discussed our findings against that paper instead-

The last sentence in paragraph 3 is incomplete. Authors should kindly resolve this since it was addressing an important point

*Authors response: I do not find the incomplete sentence.*

Conclusion
In the conclusion, authors report no association between depression levels and albuminuria and vice versa. However, authors failed to provide guidance on the next action or way forward.

Authors response: We have added this future way forward:

*“Future studies should investigate whether other depression measurements yields different results and whether eGFR is a better biomarker for future depression than albuminuria.” (Conclusion, page 28)*

6. PLOS authors have the option to publish the peer review history of their article ([what does this mean?](https://eur01.safelinks.protection.outlook.com/?url=https%3A%2F%2Fjournals.plos.org%2Fplosone%2Fs%2Feditorial-and-peer-review-process%23loc-peer-review-history&data=05%7C01%7Clise.t.gustad%40nord.no%7C26071181d4d34ed3836f08da5f204b82%7Cfed13d9f21df485d909a231f3c6d16f0%7C1%7C1%7C637926889695427629%7CUnknown%7CTWFpbGZsb3d8eyJWIjoiMC4wLjAwMDAiLCJQIjoiV2luMzIiLCJBTiI6Ik1haWwiLCJXVCI6Mn0%3D%7C3000%7C%7C%7C&sdata=BLvz49H0USS5oAgN2cz9GWbOd3MYivByGH3HPpfwpXc%3D&reserved=0)). If published, this will include your full peer review and any attached files.


If you choose “no”, your identity will remain anonymous but your review may still be made public.


**Do you want your identity to be public for this peer review?** For information about this choice, including consent withdrawal, please see our [Privacy Policy](https://eur01.safelinks.protection.outlook.com/?url=https%3A%2F%2Fwww.plos.org%2Fprivacy-policy&data=05%7C01%7Clise.t.gustad%40nord.no%7C26071181d4d34ed3836f08da5f204b82%7Cfed13d9f21df485d909a231f3c6d16f0%7C1%7C1%7C637926889695427629%7CUnknown%7CTWFpbGZsb3d8eyJWIjoiMC4wLjAwMDAiLCJQIjoiV2luMzIiLCJBTiI6Ik1haWwiLCJXVCI6Mn0%3D%7C3000%7C%7C%7C&sdata=hVQGOWuEDlwjFVqEvG9ACSMbSE0HNzoRgaia77sRAv4%3D&reserved=0).

Reviewer #1: No

Reviewer #2: **Yes:** David Nana Adjei
